# Mapping Implementation Strategies to Address Barriers to Pre-Exposure Prophylaxis Use Among Women Through POWER Up (Pre-Exposure Prophylaxis Optimization Among Women to Enhance Retention and Uptake): Content Analysis

**DOI:** 10.2196/59800

**Published:** 2024-11-15

**Authors:** Amy K Johnson, Samantha A Devlin, Maria Pyra, Eriika Etshokin, Kelly Ducheny, Eleanor E Friedman, Lisa R Hirschhorn, Sadia Haider, Jessica P Ridgway

**Affiliations:** 1 The Potocsnak Family Division of Adolescent & Young Adult Medicine Ann & Robert H Lurie Children's Hospital of Chicago Chicago, IL United States; 2 Department of Pediatrics Feinberg School of Medicine Northwestern University Chicago, IL United States; 3 Section of Infectious Diseases and Global Health Department of Medicine University of Chicago Chicago, IL United States; 4 Department of Medical Social Sciences Feinberg School of Medicine Northwestern University Chicago, IL United States; 5 Howard Brown Health Chicago, IL United States; 6 Division of Family Planning Department of OB/GYN Rush University Chicago, IL United States

**Keywords:** pre-exposure prophylaxis, PrEP, Consolidated Framework for Implementation Research, CFIR, Expert Recommendations for Implementing Change, ERIC, implementation science, HIV prevention, AIDS, United States, Black women, women’s health

## Abstract

**Background:**

Black cisgender women (hereafter referred to as “women”) experience one of the highest incidences of HIV among all populations in the United States. Pre-exposure prophylaxis (PrEP) is an effective biomedical HIV prevention option, but uptake among women is low. Despite tailored strategies for certain populations, including men who have sex with men and transgender women, Black women are frequently overlooked in HIV prevention efforts. Strategies to increase PrEP awareness and use among Black women are needed at multiple levels (ie, community, system or clinic, provider, and individual or patient).

**Objective:**

This study aimed to identify barriers and facilitators to PrEP uptake and persistence among Black cisgender women and to map implementation strategies to identified barriers using the CFIR (Consolidated Framework for Implementation Research)-ERIC (Expert Recommendations for Implementing Change) Implementation Strategy Matching Tool.

**Methods:**

We conducted a secondary analysis of previous qualitative studies completed by a multidisciplinary team of HIV physicians, implementation scientists, and epidemiologists. Studies involved focus groups and interviews with medical providers and women at a federally qualified health center in Chicago, Illinois. Implementation science frameworks such as the CFIR were used to investigate determinants of PrEP use among Black women. In this secondary analysis, data from 45 total transcripts were analyzed. We identified barriers and facilitators to PrEP uptake and persistence among cisgender women across each CFIR domain. The CFIR-ERIC Implementation Strategy Matching Tool was used to map appropriate implementation strategies to address barriers and increase PrEP uptake among Black women.

**Results:**

Barriers to PrEP uptake were identified across the CFIR domains. Barriers included being unaware that PrEP was available (characteristics of individuals), worrying about side effects and impacts on fertility and pregnancy (intervention characteristics), and being unsure about how to pay for PrEP (outer setting). Providers identified lack of training (characteristics of individuals), need for additional clinical support for PrEP protocols (inner setting), and need for practicing discussions about PrEP with women (intervention characteristics). ERIC mapping resulted in 5 distinct implementation strategies to address barriers and improve PrEP uptake: patient education, provider training, PrEP navigation, clinical champions, and electronic medical record optimization.

**Conclusions:**

Evidence-based implementation strategies that address individual, provider, and clinic factors are needed to engage women in the PrEP care continuum. Tailoring implementation strategies to address identified barriers increases the probability of successfully improving PrEP uptake. Our results provide an overview of a comprehensive, multilevel implementation strategy (ie, “POWER Up”) to improve PrEP uptake among women.

**International Registered Report Identifier (IRRID):**

RR2-10.1371/journal.pone.0285858

## Introduction

Pre-exposure prophylaxis (PrEP) is a safe and effective medication for those at risk for HIV infection. However, there are significant disparities by race and by sex in PrEP use for HIV prevention in the United States. In 2021, the PrEP-to-need ratio (number of PrEP prescriptions divided by number of new HIV diagnoses) for cisgender women (hereafter referred to as “women”) was less than half of that for men (4.1 vs 11.2), indicating substantial inequity in PrEP use among women compared with their need [1]. In particular, Black women experience one of the highest incidences of HIV among all US populations, second only to men who have sex with men (MSM) [2]. Although they constitute just 13% of the national female population, Black women account for 60% of new HIV infections among women [2].

Despite representing 19% of new HIV infections annually and 23% of all people living with HIV in the United States, women are frequently overlooked in HIV prevention efforts [1]. Challenges in providing PrEP to women include difficulty identifying those most vulnerable to HIV, low self-perception of HIV risk, low PrEP knowledge among both providers and women, and logistical barriers to PrEP initiation (eg, health care access and insurance status) [3]. Previous studies have identified successful interventions to increase PrEP uptake among MSM and transgender women, yet less is known about improving PrEP uptake among women. Potential implementation strategies that have been recommended for women include tailoring PrEP education content to women, offering PrEP at various clinics that are frequently visited by women (ie, family planning and obstetrics and gynecology), and improving the trust and communication between patients and providers [4-7]. Given the 2021 PrEP guidance to inform all sexually active patients of PrEP and recent US Food and Drug Administration approval of cabotegravir injections (ie, injectable PrEP), it is essential that health systems implement and maintain effective, evidence-based strategies to increase PrEP uptake among women [8,9].

Implementation science provides relevant conceptual and measurement frameworks to guide an assessment of factors that influence implementation outcomes. It is a particularly useful field for identifying barriers and facilitators to intervention success. The Consolidated Framework for Implementation Research (CFIR) includes 5 interrelated domains: intervention characteristics, outer setting, inner setting, characteristics of individuals involved, and the process of implementation, with several constructs in each domain [10]. As a framework, the CFIR domains allow for documenting and understanding a nonlinear process of implementation and incorporates consideration of adaptation that is often required for different contexts. Once barriers and facilitators are identified, implementation strategies can be developed to leverage facilitators and address barriers in order to achieve desired health outcomes. In addition, the CFIR has been used as a guiding framework for several studies among health care staff and patients specifically related to identifying facilitators and barriers of PrEP implementation in various health care settings across different patient populations [11-15]. However, there still remains a scarcity of studies specifically examining determinants regarding implementation of PrEP among women.

Using secondary data, the purpose of this paper is to identify barriers and facilitators to PrEP uptake and persistence among cisgender women at Howard Brown Health (HBH), a federally qualified health center (FQHC) providing LGBTQ (lesbian, gay, bisexual, transgender, and queer/questioning)-centered primary care; and map implementation strategies to identified barriers using the CFIR-ERIC (Expert Recommendations for Implementing Change) Implementation Strategy Matching Tool [16-18].

## Methods

### Overview

We conducted a secondary data analysis of previous work completed by our multidisciplinary team of HIV specialists, implementation scientists, infectious disease physicians, and epidemiologists. Multiple qualitative studies were designed to identify barriers and facilitators to PrEP uptake and persistence among women in the Midwest. HBH was selected as the primary site for these studies because they see a substantial proportion of women who could benefit from PrEP; a previous study has indicated that up to 23% of women who receive care at HBH have indications for PrEP [19]. Our studies included data collected directly from patients and providers, in both focus groups and informant interviews. Across 4 different studies, our team conducted the following: 43 individual qualitative interviews with medical providers [20], PrEP-experienced women (ie, women who had taken PrEP) [21,22], and women newly diagnosed with HIV [23]; and 2 focus groups with 16 PrEP-naïve women [21]. Each study obtained institutional review board (IRB) approval before enrolling participants in the study and maintained approval for data analysis (refer to Multimedia Appendix 1 for full IRB, study timeline, and methods review).

Full methods and results of previous studies have been published in detail elsewhere [20-23]. Both active and passive recruitment strategies were used to identify potential participants. Passive recruitment involved posting flyers within the FQHC and sending information about the study through listservs to providers and text messages to patients. Interested individuals then contacted the study team through email, text message, or phone call. Purposive sampling occurred in studies that enrolled specific patients (ie, women who were recently diagnosed with HIV). The study team would call eligible participants, inform them of the study, and schedule an interview time if interested. Convenience sampling also occurred across studies, with the research team approaching potential participants in the waiting area of the health center. Patients were informed of the study and asked if they would be interested in scheduling an interview or focus group. All participants provided informed consent before being enrolled in the respective study.

In brief, semistructured interview guides were used by facilitators with qualitative expertise to illicit barriers and facilitators to PrEP uptake and persistence among women. Provider interview guides contained questions and prompts about bringing up HIV prevention in patient encounters (“How do you introduce PrEP to women?”), experiences prescribing PrEP (“Tell me about your role in ensuring once a woman wants to start PrEP, she is able to…”), needed clinical supports and any training experienced or desired (“What additional resources are needed for more providers to offer women PrEP?”). Interviews with PrEP-experienced women contained questions about their experience being offered PrEP, taking PrEP (“What was it like starting PrEP? Did anyone from the clinic help get you started? What supports were offered?”), and either persisting on PrEP or decision making around stopping (“Were there any personal issues that made it hard to stay on PrEP? What could help you stay on PrEP?”). Interviews with women newly diagnosed with HIV contained questions about their experiences before becoming diagnosed, probing for opportunities they could have been provided PrEP (“Before you were diagnosed, were you ever offered PrEP? What do you think about PrEP as an option to prevent HIV?”). Interviews with women who were PrEP-naïve contained questions about PrEP knowledge and attitudes (“Have you ever heard of PrEP? If so, what have you heard about it? If a clinic doctor told you that you were eligible for PrEP, how would that make you feel?”) as well as barriers and facilitators to uptake. Focus groups and key informant interviews took place in a private area within the FQHC or another location that was convenient for the participant or remotely over the phone or a secure video conferencing platform (ie, Zoom [Zoon Video Communications]). Discussions lasted 30-60 minutes. All discussions were audio recorded and professionally transcribed.

For this secondary data analysis, data from 45 total transcripts were imported into Dedoose. Two coders analyzed the transcripts, and excerpts were coded into barriers or facilitators across each CFIR domain. Next, the study team mapped each main barrier or facilitator to ERIC implementation strategies. A consensus-based process was used in which the coding team presented the mapping to the larger study team for comment and discussion, consensus was reached through careful review of the data and discussion on selection of the appropriate ERIC strategy.

### Ethical Considerations

This study was reviewed by the Institutional Review Board at Ann & Robert H. Lurie Children's Hospital of Chicago and received a non-human subjects research determination (IRB2025-7451). All previous studies referenced as part of this analysis were reviewed and approved by the IRBs at the University of Chicago, Northwestern University, Ann and Robert H. Lurie Children’s Hospital of Chicago; Howard Brown Health, respectively. Verbal informed consent was obtained from participants for each study. In addition, written informed consent was obtained from participants in the study that involved accessing their electronic medical record (EMR). Participants were informed that their data would be stored with no identifying information and used to understand PrEP use in women. Participants were also informed that their deidentified data would be shared across institutions for research and dissemination purposes. Participants were compensated US $40-50 across studies by a physical gift card or cash by an electronic payment app. Data within this analysis do not include any identifying information.

## Results

Our studies centered women’s and providers’ experiences and identified both barriers and facilitators to PrEP uptake across the CFIR domains. The CFIR domains included intervention characteristics, specifically characteristics of PrEP that impact uptake, such as burden of daily dosing, cost concerns, and need for multiple clinic visits; inner setting included the FQHC environment and staff, such as routine PrEP education and use of the EMR to aid prescribing PrEP; outer setting encompassed the setting in which the inner setting exists, essentially the PrEP research and policy environment; and characteristics of individuals that impact uptake included self-efficacy, PrEP knowledge, and attitudes toward PrEP. Across the studies that involved cisgender women, 56%-100% identified as Black or African American [21-23].

In our interviews with Black women at HBH who either discontinued or persisted on PrEP, participants consistently stressed the importance of ensuring women are aware of PrEP and receive education about it from trusted health care providers [22]. This participant highlighted the importance of providers offering PrEP without judgment to women.

I think that [PrEP] message needs to get out there without it being judgmental. Like I said, my experience with Dr. [name redacted] shaped my whole perspective…So, for me, it was just [my doctor] being understanding that I wanna protect myself, I wanna protect my kids and my family, the future of my family, and I’m just thankful that I had options and means, and I just wish that all women had that, as well.

Many of these patients reported discontinuing PrEP when they were considering getting pregnant because they believed PrEP would cause harmful effects.

And then, too, with trying to conceive, it’s like I did not want to chance it happening again, and it seems like whatever it was, you know my body – even though it’s been like almost two – a year-and-a-half [since stopping PrEP], it seems like it took a while for that [PrEP] kinda finally get out of my system after taking it for so many years.

Similarly, we learned during interviews with women who had been recently diagnosed with HIV that PrEP education could be a potential way to overcome the stigma associated with PrEP use among women and to get more women on PrEP [23]. We also discussed delivering PrEP education to patients in our interviews with providers from HBH [20]. They emphasized how PrEP education is given to essentially anyone who is HIV-negative and is coming in for routine sexually transmitted infection (STI) testing and how PrEP pamphlets are readily available for patients.

... nursing and health educators present PrEP to every patient, just so that people are aware. And then, people can decide if it’s something that’s good for them and their bodies and their health. But just to sort of get the messaging out is super important.

However, some providers noted differences in how they present PrEP information to cisgender women versus MSM and transwomen, including discussing how most PrEP studies have been conducted with MSM and transwomen and highlighting how on-demand PrEP counseling cannot be offered to cisgender women. Providers emphasized the need for PrEP training to specifically engage in culturally tailored conversations with women.

I also think ongoing education for the people that are interfacing with clients about how to assess…offering PrEP to anybody who’s sexually active, and not really getting in the weeds with like who is at risk or who is not, but just with offering it...ongoing educational opportunities for providers would be good.

Thus, implementation strategies that emerged include routine patient education and standardized provider training which address barriers across the intervention characteristics, characteristics of individuals, and inner setting domains of CFIR using multiple ERIC strategies including developing educational materials, conducting standardized routine training, making training dynamic, preparing patients to be active participants, and providing feedback. As one participant stated, it is important for training to be standardized and ensure all team members receive the same information.

I think that if you implement a training like this...it's more important for me to have a checkpoint within the systems that I use to say that this person has been counseled on PrEP and to say that everyone that touches that patient when they come in understands that they've gotten that information and are able to kind of build upon that information. Because it makes no sense for me to able to give a person an overview of what PrEP is, how PrEP can be effective, and how PrEP can work for them, but they go to the second person and the second person just looks at them and says, ‘I don’t know what you're talking about.’

A key theme from provider interviews was the importance of EMR clinical decision support for PrEP, particularly clearer guidance on indications for PrEP for women. Providers noted that a best practice advisory alert or similar feature would likely enable them to facilitate PrEP prescription among women [20]. Providers at HBH discussed how even though their EMR was well designed, there were still inconsistencies and some difficulties with using it to document HIV risk factors among women. One provider explained documenting PrEP within the EMR required navigating multiple screens and that it would be easier if there was a quicker option within the EMR.

I wish that that part [PrEP documentation] of the [EMR] was easier to use. I would use it if it was easier to use and change and modify, and it wasn’t like a – seeming like a four- or five-step process…It’s easier for me to do what we call like a dot raise or a quick text.

Similarly, in our study of women recently diagnosed with HIV, we discovered that many women who were engaged in the health care system were not offered PrEP as an HIV prevention tool before their diagnosis. Many of the characteristics known to increase vulnerability to HIV were not documented in the EMR (eg, male partner’s sexual activity with other men, male partner’s incarceration history, and previous STI diagnoses) but only elucidated through qualitative interviews [23]. Thus, to leverage the facilitator of the inner setting (EMR reminders) and address barriers related to intervention characteristics (required dosing, patient identification, and prescription), EMR optimization emerged as an implementation strategy using the ERIC strategies of reminding clinicians, using data experts, facilitating relay of clinical data to providers, and centralizing technical assistance.

In our interviews with HBH providers, the importance of PrEP navigation was consistently highlighted as a facilitator for PrEP initiation and persistence. Providers also noted that PrEP navigators help to address barriers that may cause women to discontinue PrEP or fall out of care, including giving medication adherence reminders, getting health PrEP insurance coverage for clients, and working with them to solve logistical challenges such as unemployment or changes in their schedule [20]. For example, this provider noted how the PrEP navigation team helps support patients.

We have a PrEP Navigator Team, so if anyone comes in saying, you know, ‘I had a problem with my PrEP or insurance issue,’ I can just leave a note on the PrEP Navigator desktop saying, ‘Hey can you reach out to this person for more information?’ So…all I have to do is prescribe it. Someone else is gonna figure out the roadblocks and the hurdles.

In addition to providers, interviews with Black women from HBH who had either discontinued or persisted on PrEP also revealed the importance of PrEP navigation for helping with their initiation and adherence. Many patients highlighted how PrEP navigators specifically helped with getting the cost of PrEP covered and eliminating barriers to initiation [22]. PrEP navigation as an implementation strategy addresses barriers at both the inner and outer setting by using ERIC strategies of tailoring strategies and creating new clinical teams.

A theme that emerged from our preliminary studies at the inner CFIR level was the importance of institutional support, clinical infrastructure, and the influence of a PrEP advocate on staff. Providers noted the benefit of having an experienced colleague on site to answer questions and serve as an advocate for PrEP, which increased their comfort with prescribing PrEP. Clinical champions are individuals within an organization who advocate for a particular change, collaborate with others, and use their knowledge and skills to facilitate adoption and implementation of an intervention. Clinical champions can work at different levels within an organization and across sites to support successful implementation. Clinical champions as an implementation strategy to improve PrEP uptake leverages existing strengths and provider knowledge and uses ERIC strategies of facilitation, identifying champions, identifying early adopters, and capturing and sharing knowledge.

Leveraging these results, the POWER Up (PrEP Optimization among Women to Enhance Retention and Uptake) strategies were created for engaging women in the PrEP care continuum at HBH. Strategies include both provider-centered strategies, as well as health system-centered strategies. The POWER Up strategies include (1) routine PrEP education, (2) standardized provider training, (3) EMR optimization, (4) PrEP navigation, and (5) PrEP clinical champions (Table 1).

**Table 1:** Pre-exposure prophylaxis optimization among women to enhance retention and uptake implementation strategies and corresponding levels, determinants, and mechanisms of change.

POWER Up^a^ strategy		CFIR^b^ level	Implementation barrier or facilitator	ERIC^c^ strategies	Mechanism of change
**Routine patient education**					
	Develop a guide for delivering PrEPd education by discussions during appointments Develop brochures and pamphlets with specific considerations for Black cisgender women	Inner setting Characteristics of individuals	Patient knowledge deficit (-) Patient beliefs toward HIV vulnerability (-)	Develop educational materials Prepare patients to be active participants Intervene with patients to enhance uptake and adherence Conduct ongoing training	Awareness- building Knowledge-acquisition Self-efficacy
**Provider training**					
	Develop training sessions for providers to complete that include providing patient education; identifying PrEP-eligible women; how to offer PrEP; how to work with PrEP navigation team; practicing difficult conversations with digital Black cisgender female patients (avatars and role play)	Intervention characteristics Inner setting Characteristics of individuals	Provider knowledge deficit (-) Competing clinical demands (-) Provider low comfort level in discussing PrEP (-)	Provide clinical supervision Audit and provide feedback Conduct ongoing training Make training dynamic	Awareness-building Knowledge-acquisition Self-efficacy
**PrEP^d^ navigation**					
	Determine staff who will be designated as PrEP navigators Develop model to delineate roles and responsibilities of PrEP navigators to meet with patients to support initiation and monthly check-ins to support persistence thereafter (appointments, refill reminders based on patients’ own schedule)	Inner setting Outer setting	Turnover of staff (-) Limited staff to support PrEP uptake (-) Clinic programs (+) Integrating services (+) Clinic visits (-) Laboratory results (-)	Tailor strategies Create new clinical teams	Capacity building Real-time training and consultation
**EMR^e^ optimization**					
	Assess existing EMRe software and templates for documenting HIV risk factors Implement clinical decision support tools for identification of PrEP candidates and prescription of PrEP Develop and incorporate PrEP indication documentation into the EMR Provide regular electronic reports of PrEP care continuum outcomes for clinic staff to review and act upon	Intervention characteristics Inner setting	Dosing (-) Prescription (-) EMR reminders (+)	Facilitate relay of clinical data to providers Remind clinicians Centralize technical assistance Use data experts	Capacity building Streamline processes Real-time training and consultation
**Clinical champions**					
	Determine staff who will be designated as clinical champions through early adopters Communicate to clinic staff who the champions are and identify their role to support facilitation of PrEP uptake (clinical innovation)	Inner setting Characteristics of individuals	Clinic programs (+) Knowledge (-/+)	Facilitation Identify and prepare champions Identify early adopters Capture and share local knowledge Use an implementation advisor Use “train the trainer” strategies	Real-time training and consultation Support knowledge and skill gain of staff

^a^POWER Up: PrEP Optimization among Women to Enhance Retention and Uptake.

^b^CFIR: Consolidated Framework for Implementation Research.

^c^ERIC: Expert Recommendations for Implementing Change.

^d^PrEP: pre-exposure prophylaxis.

^e^EMR: electronic medical record.

## Discussion

### Principal Findings

Our collective qualitative studies identified intervention opportunities and implementation strategies for improving PrEP uptake among Black women, which we refer to as POWER Up [24]. There has been a recent call to improve PrEP implementation for uptake among women through increased awareness and knowledge, better access to health care, and provider cultural and gender competency [25,26]. By addressing multiple levels including the individual (patient education), provider (provider training), clinic system (EMR tools and clinical champions), and broader health system (PrEP navigation), the proposed POWER Up strategies provide a comprehensive implementation plan that is likely to have a greater impact than any single strategy alone for increasing PrEP uptake [27-29].

The results highlight the need for patients to receive education on assessing their own HIV risk and HIV prevention options. Based on our interviews with providers and patients, patient education should consist of comprehensive information about PrEP (eg, effectiveness and side effects), as well as specific considerations for women. Others have advocated for similar tailoring of PrEP educational tools for women, while simultaneously ensuring providers have the confidence and competency to discuss PrEP with women who are vulnerable to HIV, particularly Black women [30-33]. Providers should deliver PrEP education to all sexually active women and have readily available brochures or pamphlets. These pamphlets should include visuals that appeal to women and information that may be specific to women, including information about taking PrEP during pregnancy and breast feeding. In addition, comprehensive and culturally-competent provider-patient communication has been noted as a critical element to improve PrEP engagement among women [34-36].

Previous research has shown that providers serve as an important gateway to introducing PrEP to patients, yet they are often uncertain about how to assess HIV risk and indications for PrEP, specifically for Black women [37-40]. Similarly, we found providers to be generally comfortable discussing PrEP with their patients but having some concerns talking about PrEP with women of color. To address this barrier, providers recommended role-playing be included within trainings about PrEP. Indeed, an effective strategy for increasing provider rates of prescribing PrEP for women are digital interactional training sessions [41]. We recommend including role-playing and interactive components in provider PrEP trainings so that providers can practice having these important conversations with patients. We also recommend including a supplementary handout so that providers have readily available information when counseling or prescribing PrEP. Updates to clinical guidelines should be disseminated to providers as needed and a feedback loop should be established to offer assessment of providers’ application of the training. Furthermore, the Centers for Disease Control and Prevention advises that all patients who are sexually active receive PrEP counseling.

Intervention studies have demonstrated that use of the EMR to provide clinical decision support for PrEP and to report PrEP outcomes to providers is an important tool for improving uptake [42-44]. However, few studies have analyzed its use in the context of assessing HIV/STI risk [45,46]. Preliminary models have primarily relied on structured EMR fields including diagnosis codes and laboratory tests that capture a limited amount of information regarding HIV risk factors [47-49]. Other relevant information that could be helpful in predicting HIV risk among women, including explicit details of social and behavioral determinants such as sexual orientation and sexual activity, are typically collected in a narrative or seminarrative format through free-text notes within the social history section of the EMR [50,51]. Consequently, fields with risk factors included within EMR alerts to identify women at high risk for HIV are often limited or incomplete, meaning EMR alerts to counsel patients and prescribe PrEP are not widely used or are inaccurate among women.

Our team identified clear gaps in documenting relevant relationship and contextual information within the EMR that impacts a woman’s HIV risk, and we identified both missed opportunities for prescribing PrEP to women as well as ways we can better capture data in the EMR to guide automated alerts of PrEP indications among women. These results can be used to optimize the EMR by creating a comprehensive sexual or social history form that considers HIV risk factors specific to women, including sexual activity of their partners. These standardized prompts and templates can be adapted to health centers as needed such that HIV risk, PrEP discussion or education, PrEP initiation, and PrEP adherence are all adequately documented and can be queried. EMR optimization allows health systems to track not only PrEP outcomes, but also to document barriers and facilitators among women at each stage of the PrEP care continuum.

Finally, PrEP navigation has been shown to improve PrEP uptake and persistence in diverse patient populations [52-54]. Among women, the results of PrEP navigation are mixed, with few studies specifically analyzing the impact of navigation on PrEP care continuum outcomes among women. A pilot study involving a randomized controlled trial compared an intervention of counseling and navigation with a brief information session for increasing PrEP among cisgender women. Although PrEP knowledge was higher among the group that received counseling and navigation, there were no significant differences seen in PrEP initiation by study arm [55]. However, another study showed that among Black and Latina cisgender women, PrEP initiation was significantly associated with receiving benefits navigation services [56]. It is likely that PrEP navigation services for women needs to be more comprehensive when compared with the model of navigation that has been used for other populations. At HBH, PrEP navigators screen and link patients interested in PrEP, including patients receiving walk-in STI testing, and assist in linking them to PrEP-related financial supports. Patients can also be linked to social services and are followed up for the first three months to assist with adherence and follow-up appointments. PrEP navigators also contact select patients who appear to be at risk for falling out of care every month to provide additional PrEP support. We recommend PrEP navigation should consist of at least 1 person who is able to assist with insurance costs related to PrEP initiation, as well as persistence efforts, including appointment and adherence reminders. PrEP navigators should respond to messages as needed and conduct at least monthly check-in appointments with patients who are currently on PrEP for the first 6 months of persistence.

### Limitations

This study has a few limitations to acknowledge. Data were collected from providers and patients in 1 large Midwest city and therefore may not reflect the experiences of providers and patients from other regions of the United States, specifically, regions with differences in the HIV epidemic and PrEP implementation. However, as our intention is to implement POWER Up within the Midwest first and to disseminate lessons learned from implementation, we specifically chose to analyze data from this region. Furthermore, we were not able to validate our findings by presenting them to participants for feedback. To our knowledge, we did not have any repeat participants across studies, yet we may have included some participants in multiple studies unknowingly. Although this does not change our findings, it may indicate that women who are engaged in the health care system were more likely to participate in our research studies, and the proposed implementation strategies may not address barriers among women who are less engaged in the health care system. Our study team included clinicians; however, we were not able to document patient feedback on implementation strategies. Future studies should query both patients and providers for their feedback on the proposed implementation strategies, specifically probing for adaptations by local context.

### Conclusions

HIV prevention efforts continue to overlook the needs of women, especially Black cisgender women. Health systems must adopt evidence-based implementation strategies that address individual, provider, and clinic factors. The POWER Up strategies provide a comprehensive implementation plan that may lead to increased PrEP awareness, uptake, and persistence among women. By implementing the POWER Up strategies, health centers can find opportunities for engaging women in the PrEP care continuum and improve uptake and persistence of PrEP as needed.

## Appendix

Multimedia Appendix 1 Additional information for previous studies.

## Abbreviations

CFIR: Consolidated Framework for Implementation Research
EMR: electronic medical record
ERIC: Expert Recommendations for Implementing Change
FQHC: federally qualified health center
HBH: Howard Brown Health
IRB: institutional review board
LGBTQ: lesbian, gay, bisexual, transgender, and queer/questioning
MSM: men who have sex with men
POWER Up: POWER Up Optimization among Women to Enhance Retention and Uptake
PrEP: pre-exposure prophylaxis
STI: sexually transmitted infection

## Data Availability

The datasets analyzed during this study are available from the corresponding author upon reasonable request.
