# Supplementary material for: Mapping Implementation Strategies to Address Barriers to Pre-Exposure Prophylaxis Use Among Women Through POWER Up (Pre-Exposure Prophylaxis Optimization Among Women to Enhance Retention and Uptake): Content Analysis
Source: JMIR Form Res. 2024 Nov 15;8:e59800. doi: 10.2196/59800 (PMC11607547; doi:10.2196/59800)
Supplement: Multimedia Appendix 1 [file formative_v8i1e59800_app1.docx]

Multimedia Appendix 1

| Publication Title | Study IRB and Approval Number | Description of Methods | Recruitment Period |
| --- | --- | --- | --- |
| Black Cisgender Women's PrEP Knowledge, Attitudes, Preferences, and Experience in Chicago | University of Chicago (IRB18-0390) | Two focus groups were conducted with PrEP-naïve women, and interviews were conducted with 7 PrEP-experienced women. Data were analyzed thematically. | April 2018 to August 2018 |
| Provider Perspectives on Factors Affecting the PrEP Care Continuum Among Black Cisgender Women in the Midwest United States: Applying the Consolidated Framework for Implementation Research | University of Chicago (IRB19-1045) | We conducted semi-structured interviews, guided by the Consolidated Framework for Implementation Research (CFIR), with 10 medical providers at agencies/clinics to identify PrEP implementation strategies for Black cisgender women in the Midwestern United States. Data were analyzed thematically. | December 2019 to February 2020 |
| HIV Pre-exposure Prophylaxis Use and Persistence among Black Ciswomen: “Women need to protect themselves, period” | University of Chicago (IRB19-1045) | We conducted interviews with 8 Black women at Howard Brown Health (HBH) who had either persisted on PrEP for at least 6 months or who had discontinued PrEP. Data were analyzed thematically. | February 2020 to June 2020 |
| Evaluation of Multiple Data Sources for Predicting Increased Need for HIV Prevention among Cisgender Women: Understanding Missed Opportunities for Pre-exposure Prophylaxis (PrEP) | University of Chicago (IRB19-1345) | We conducted semi-structured interviews with 18 ciswomen living with HIV who received care at either the University of Chicago or HBH. Data were analyzed thematically for common factors related to potential pre-diagnosis intervention points and missed opportunities for PrEP. | November 2019 to April 2021 |
